# Development and application of a low-priced duplex quantitative PCR assay based on SYBR Green I for the simultaneous detection of porcine deltacoronavirus and porcine sapelovirus

**DOI:** 10.17221/79/2022-VETMED

**Published:** 2023-03-23

**Authors:** Si-Jia Lu, Meng-Yao Ma, Xiao-Guang Yan, Fu-Jie Zhao, Wen-Yang Hu, Qing-Wen Ding, Hao-Jie Ren, Yu-Qiang Xiang, Lan-Lan Zheng

**Affiliations:** College of Veterinary Medicine, Henan Agricultural University, Zhengzhou, Henan, P.R. China

**Keywords:** duplex real-time qPCR, PDCoV, PSV

## Abstract

Porcine deltacoronavirus (PDCoV) and porcine sapelovirus (PSV) are two viruses that can cause diarrhoea in pigs and bring great economic loss to the pig industry. In this research, a duplex real-time quantitative polymerase chain reaction (qPCR) assay based on SYBR Green I was developed to simultaneously detect PDCoV and PSV. No specific melting peaks were found in other porcine diarrhoea-associated viruses, indicating that the method developed in this study had good specificity. The detection limits of PDCoV and PSV were 1.0 × 10^1^ copies μl^–1^ and 1.0 × 10^2^ copies μl^–1^, respectively. The duplex real-time qPCR assay tested two hundred and three (203) intestinal and faecal samples collected from diarrhoeal and asymptomatic pigs. The positive rates of PDCoV and PSV were 20.2% and 23.2%, respectively. The co-infection rate of PDCoV and PSV was 13.8%. To evaluate the accuracy of the developed method, conventional PCR and singular TaqMan real-time qPCR assays for PDCoV/PSV were also used to detect the samples. The results showed that the duplex real-time qPCR assay was consistent with the singular assays, but its sensitivity was higher than conventional PCR methods. This duplex real-time qPCR assay provides a rapid, sensitive and reliable method in a clinic to simultaneously detect PDCoV and PSV.

Porcine deltacoronavirus (PDCoV) is an enveloped positive-stranded RNA virus with a genome of 25.4 kb which encodes four key structural proteins: membrane (M), envelope (E), spike (S) and nucleocapsid (N), among which the *S* gene is an important virulence gene and its mutation is likely to lead to cross-species transmission (Lee and Lee 2014; Song et al. 2015; Li et al. 2018). In 2012, Woo et al. (2012) discovered a novel coronavirus from pigs in Hong Kong and classified it in the *Deltacoronavirus* genus. PDCoV was first detected in a pig farm in Ohio in the USA with an outbreak of diarrhoea in 2014 (Wang et al. 2014). PDCoV has been discovered in the USA, China, South Korea, Vietnam, and many other countries, causing great financial losses to the global pig industry (Lee and Lee 2014; Wang et al. 2014; Le et al. 2018; Zhang et al. 2019). PDCoV can infect pigs of different ages and cause clinical symptoms such as diarrhoea, vomiting, and dehydration (Li et al. 2019a). PDCoV is mainly obtained through faecal-oral transmission, with a rapid transmission and an acute onset. The small intestine, especially the ileum and the jejunum of pigs infected by PDCoV, usually has obvious pathological changes such as intestinal wall thinning and mucosal shedding (Chen et al. 2015). Besides pigs, PDCoV can infect poultry (Liang et al. 2019; Boley et al. 2020), humans (Lednicky et al. 2021) and sparrows, which are now considered to be important intermediate hosts (Ye et al. 2020).

Porcine sapelovirus (PSV) is a non-enveloped, positive-stranded RNA virus with a spherical viral particle that is around 25–30 nm in diameter and belongs to the genus of *Sapelovirus*, in the *Picornaviridae* family. The genome length of PSV is about 7.5 kb with a 5' untranslated region (UTR), one open reading frame (ORF) encoding four structural proteins (VP1 to VP4) and seven non-structural proteins (2A to 2C, 3A to 3D), a 3' UTR, and a poly(A) tail (Boros et al. 2020). PSV was first discovered in the UK in 1958 (Lamont and Betts 1960), and since then it has spread all over the world (Donin et al. 2014; Son et al. 2014; Arruda et al. 2017; Masuda et al. 2018; Li et al. 2019b).

PSV can cause poliomyelitis, diarrhoea, pneumonia, reproductive disorders, respiratory distress and other symptoms in pigs (Son et al. 2014; Arruda et al. 2017; Masuda et al. 2018; Li et al. 2019b; Harima et al. 2020), where the main transmission pathway is the faecal-oral route, which has a significant impact on the development of the global pig industry.

For a clinical diagnosis, it is difficult to differentiate PDCoV and PSV accurately by the symptoms alone, since both of the viruses can cause diarrhoea in piglets, and the clinical symptoms are similar. Ding et al. (2021) collected 30 intestinal samples and 62 faecal samples from piglets that died of diarrhoea to detect PDCoV and PSV. The co-infection rate of PDCoV and PSV was 13.04%, suggesting that the co-infection of PDCoV and PSV should be investigated further. Currently, there are numerous detection methods for PDCoV, including the reverse transcription polymerase chain reaction (RT-PCR), TaqMan PCR and SYBR Green I RT-PCR detection methods (Ma et al. 2018; Ding et al. 2020; Pan et al. 2020). Detection methods for PSV include RT-PCR and TaqMan PCR assays (Jiang et al. 2019; Kumari et al. 2020). However, no duplex detection method for PDCoV and PSV has been established until now. In this research, a duplex real-time quantitative polymerase chain reaction (qPCR) assay based on SYBR Green I with high efficiency, accuracy and simplicity was developed, which can be used to simultaneously detect PDCoV and PSV.

## MATERIAL AND METHODS

### Virus strains and testing specimens

PDCoV, PSV, porcine epidemic diarrhoea virus (PEDV), transmissible gastroenteritis virus (TGEV) and porcine circovirus type 2 (PCV2) were identified and preserved by the Key Laboratory for Animal-derived Food Safety of Henan Province, P.R. China.

Two hundred and three (203) intestinal and faecal samples were collected from the Henan, Shanxi, Hebei, Jiangxi and Sichuan provinces of China from 2019 to 2022. The samples were collected from diarrhoeal or asymptomatic pigs of different ages.

### Preparation of the nucleic acid templates

One hundred (100) mg of the intestinal samples and faecal swab specimens treated with aseptic procedures were first homogenised, and then diluted with phosphate-buffered saline (PBS) at a ratio of 1 : 10, and the supernatant solution was harvested by centrifugation (12 000 × *g*, 10 minutes). The viral RNA was obtained with the TRIzol reagent (Invitrogen, Carlsbad, CA, USA), and reverse transcription was conducted with a HiScript^®^ II 1^st^ Strand cDNA Synthesis Kit (Vazyme, Nanjing, P.R. China) to acquire the cDNA.

The viral DNA was obtained by using a DNA Isolation Kit (Sangon Biotech, Shanghai, P.R. China). Dulbecco’s Modified Eagle Medium (DMEM) was used as a negative control and all the extracted cDNA and DNA were stored at −80 °C for further use.

### Primer designation

The sequences of PDCoV (GenBank Accession No.: MN942260, MN173803, MH025762, MK330605, MK57280, MK355396, MG832584, MH708123, MG242062, MZ802777 and MW685624) and PSV (GenBank Accession No.: MH626635, MK497044, MN807752, MN685785, MN836683, MN685785, MK378925, MK378966, MK378953, LC425417, LC508234 and MK378966) were aligned by MegAlign software v7.1.0 (DNASTAR, Madison, WI, USA) and primers were designed on the highly conserved regions of the PDCoV *S* gene and PSV *5*' *UTR* gene by using Primer Premier v5.0 software (Premier Biosoft, Palo Alto, CA, USA). The primers and probes of the PDCoV/PSV TaqMan real-time qPCR were synthesised according to the instructions of previous studies (Zheng et al. 2019; Li et al. 2021). The information on the primers in this study is listed in Table 1, and all the primers and probes were synthesised by SanYa Biotech Henan Co. Ltd. (P.R. China).

**Table 1:** The information on the primers and probes

Methods	Primers and probes	Sequence (5' to 3')	Product size (bp)	Target gene	Reference
Duplex SYBR Green I qPCR & conventional PCR (PDCoV/PSV)	PDCoV-F1	CGTTAACCTCTTCTCACCACTT	115 bp	*S*	This study
	PDCoV-R1	GCTGAGAGTCTGGTTGGTTATT			
	PSV-F1	TGCTCCTTTGGTGATTCGG	200 bp	*5*' *UTR*	
	PSV-R1	CGACCCTATCAGGCAGTATAGA			
TaqMan qPCR (PDCoV)	PDCoV-F2	GACTCCTTGCAGGGATTATGG	90 bp	*M*	Zheng et al. (2019)
	PDCoV-R2	GCTTAACGACTGGTGTGAGAA			
	PDCoV-probe	FAM-ATGGGTACATGGAGGTGCATTCCC-TAMRA			
TaqMan qPCR (PSV)	PSV-F2	ACTTGACGAGCGTCTCTTTG	105 bp	*5*' *UTR*	Li et al. (2021)
	PSV-R2	CGACCCTATCAGGCAGTATAGA			
	PSV-probe	FAM-AGTGAGCTTCCAGGTTGGGAAACC-TAMRA-N			

PCR = polymerase chain reaction; PDCoV = porcine deltacoronavirus; PSV = porcine sapelovirus; qPCR = quantitative polymerase chain reaction; UTR = untranslated region

### Preparation of the standard recombinant plasmids

Conventional PCR assays for PDCoV and PSV were also developed in this study to obtain target fragments. The PCR reaction system contained 12.5 μl of the 2 × Taq DNA Master Mix (TaKaRa, Dalian, P.R. China), 0.5 μl (25 μM) of the forward and reverse primers, 2 μl of the cDNA template of PDCoV/PSV, and 9.5 μl of deionised water (ddH_2_O) in a 25 μl volume. The negative control was amplified with ddH_2_O as the template. The PCR was conducted with a Veriti^™^ Dx Thermal Cycler (Applied Biosystems, Waltham, MA, USA), and the parameters were as follows: pre-denaturation at 95 °C for 5 min, then 35 cycles of 95 °C for 30 s, 58 °C for 30 s, 72 °C for 60 s; 72 °C for 10 minutes. The target fragments of PDCoV and PSV were visualised and confirmed by 2.0% agarose gel electrophoresis and then recovered with a V-ELUTE Gel Mini Purification Kit (Zomanbio, Beijing, P.R. China).

After that, the recovered fragments were cloned into a pMD-18T vector (TaKaRa, Dalian, P.R. China) to construct the recombinant plasmids pMD-PDCoV and pMD-PSV. pMD-PDCoV and pMD-PSV were transferred into *Escherichia coli* DH5α cells and were then inoculated in the selective medium to isolate the positive clones. All the positive clones were confirmed by sequencing. After measuring the concentrations of pMD-PDCoV and pMD-PSV, the copy numbers were calculated and the two plasmids were then ten-fold serially diluted to construct the standard curves.

### Establishment and optimisation of the PDCoV/PSV duplex real-time qPCR assay

SYBR Green I is a non-specific nucleic acid dye that binds to dsDNA, and the fluorescence value is positively correlated with the number of amplified products. The melting curves of the real-time qPCR were constructed by constantly monitoring the fluorescence signals from 65 °C to 95 °C by 0.5 °C increments for each cycle. When the amplified products were heated to the melting temperature (Tm), the fluorescence signal would be sharply reduced. The specific melting peaks can be obtained by plotting the value of ΔF/ΔT (fluorescence change/temperature change) with the temperature, which was utilised to distinguish the PDCoV and PSV specific amplifications and eliminate interference from any non-specific amplifications.

The singular qPCR assays for PDCoV and PSV were developed by using a fluorescence qPCR instrument (CFX96^™^; Bio-Rad, Hercules, CA, USA), before optimising the duplex qPCR assay. The reaction mixture of the singular qPCR assay contained 10 μl of the 2 × ChamQ Universal SYBR qPCR Master Mix (Vazyme, Nanjing, P.R. China), 0.5 μl (25 μM) each of the forward and reverse primers, 2 μl of the positive plasmid, and ddH_2_O up to 20 μl. The thermocycling programme was pre-denaturation at 95 °C for 3 min, followed by 40 cycles of 95 °C for 10 s, and 60 °C for 30 seconds. Then, the duplex real-time qPCR assay for PDCoV/PSV was optimised by a series of experiments including the PCR reaction program parameters, primer concentrations and annealing temperatures and ddH_2_O was used as a negative control.

### Sensitivity, specificity and reproducibility analysis

The recombinant plasmids (pMD-PDCoV and pMD-PSV) were serially diluted ten-fold (from 10^9^ to 10^0^ copies μl^–1^) to determine the sensitivity of the duplex real-time qPCR for PDCoV and PSV. To confirm the specificity of this method, the cDNA/DNA extracted from PDCoV, PSV, PEDV, TGEV, and PCV2 were amplified in parallel. At the same time, we also used ddH_2_O as a negative control. The repeatability of the developed duplex real-time qPCR assay was evaluated using three different concentrations of the standard plasmids of pMD-PDCoV and pMD-PSV (10^8^ copies μl^–1^, 10^6^ copies μl^–1^, 10^4^ copies μl^–1^). The intra- and inter-batch of the detection was evaluated by calculating the coefficient of variation (CV) values in triplicate.

### Clinical application of the duplex SYBR Green I qPCR

In order to evaluate the clinical application value of the duplex SYBR Green I qPCR, we collected two hundred and three (203) clinical samples from the Henan, Shanxi, Hebei, Jiangxi and Sichuan provinces of China from 2019 to 2022. The nucleic acids were extracted and tested by the developed singular and duplex real-time qPCR assays.

### Comparison of the duplex SYBR Green I qPCR, conventional PCR and TaqMan qPCR assays

Meanwhile, 203 clinical samples were also tested using conventional PCR and singular TaqMan qPCR assays. The reaction systems and procedures for the conventional PCR were the same as that used to construct the standard recombinant plasmids. The TaqMan real-time qPCR methods for PDCoV and PSV were performed according to the methods of previous studies (Zheng et al. 2019; Li et al. 2021), and the relevant information on the primers and probes is listed in Table 1. The reaction system of the TaqMan real-time qPCR method for PDCoV is 12.5 μl of the 2 × Premix ExTaq, 1 μl of PDCoV-F2/R2 (10 μmol·l^–1^), 1 μl of a PDCoV-TaqMan probe (5 μmol·l^–1^) and 2 μl of the cDNA template, adding deionised water to 25 μl, where the reaction parameters are as follows: 95 °C for 30 s, 95 °C for 5 s, 60 °C for 30 s, at 40 cycles. The reaction system of the TaqMan real-time qPCR method for PSV is 12.5 μl of the 2 × Premix ExTaq, 0.5 μl of PSV-F2/R2 (10 μmol·l^–1^), 1 μl of the PSV-TaqMan probe (5 μmol·l^–1^), 3 μl of the cDNA template and 7.5 μl of deionised water, and the thermal cycle procedure is the same as that of the TaqMan real-time qPCR assay for PDCoV. All the positive samples identified by the real-time qPCR assays were validated by DNA agarose gel electrophoresis to ensure the correctness and reliability of the results. In addition, we randomly selected five samples for sequencing which tested positive by the duplex SYBR Green I qPCR, but tested negative by the other assays.

## RESULTS

### Optimisation of the duplex SYBR Green I real-time qPCR

**Figure 1:**
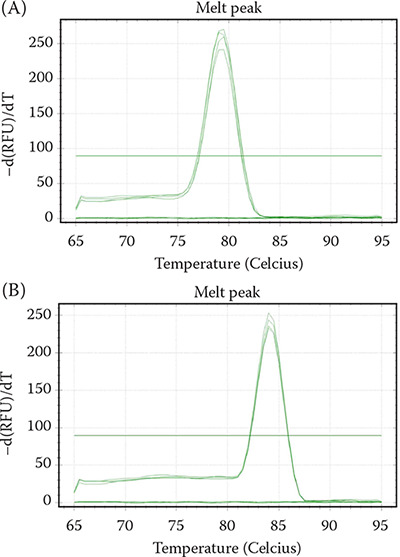
Melting curves analysis of singular real-time qPCR assay (A) Melting curves of the singular real-time qPCR assay for PDCoV. (B). Melting curves of the singular real-time qPCR assay for PSV

**Figure 2:**
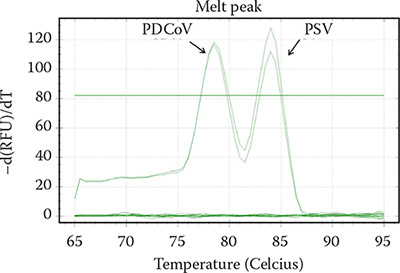
Melting curves of the duplex real-time qPCR assay

The assay was optimised with regard to the concentrations of the primers and reagents, as well as the parameters of the PCR reaction. The optimised procedure was determined as 10 μl of the 2 × ChamQ Universal SYBR qPCR Master Mix, 1.0 μl each of the cDNA template of PDCoV and PSV, 0.5 μl (25 μM) of the forward and reverse primers of PDCoV and PSV, and 6 μl of ddH_2_O. The final thermocycler parameters of the duplex real-time qPCR were as follows: pre-denaturation at 95 °C for 3 min, followed by 40 cycles of 95 °C for 10 s, 58 °C for 30 s, 72 °C for 30 seconds. The melting curves showed that the specific melting peaks for PDCoV and PSV were about 78.5 °C and 84 °C, respectively, which was consistent with that of the singular real-time qPCR assays. The results of the optimisation of the singular and duplex real-time qPCR are presented in Figure 1 and Figure 2.

### PDCoV and PSV standard curves

**Figure 3:**
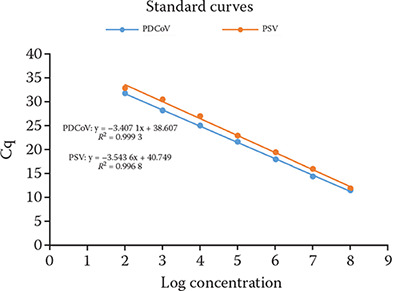
Standard curves of PDCoV and PSV

The 115 bp of the partial PDCoV-S DNA fragment and 200 bp of the partial PSV-5' UTR DNA fragment were obtained and sub-cloned to a pMD18-T vector to construct the positive plasmids. The concentrations of pMD-PDCoV and pMD-PSV were 117.5 ng μl^–1^ and 164.9 ng μl^–1^, respectively. After calculation, the copy numbers of the two standard plasmids were 3.82 × 10^10^ copies μl^–1^ for PDCoV and 5.20 × 10^10^ copies μl^–1^ for PSV. Both plasmids were diluted from 1 × 10^9^ to 1 × 10^0^ copies μl^–1^ in a series of ten-fold ratios to obtain standard curves with ddH_2_O. The standard curves of PDCoV and PSV were y = −3.407 1x + 38.607 (*R*^2^ = 0.999 3) and y = −3.543 6x + 40.749 (*R*^2^ = 0.996 8), respectively (Figure 3).

### Sensitivity of the duplex SYBR Green I real-time qPCR

**Figure 4:**
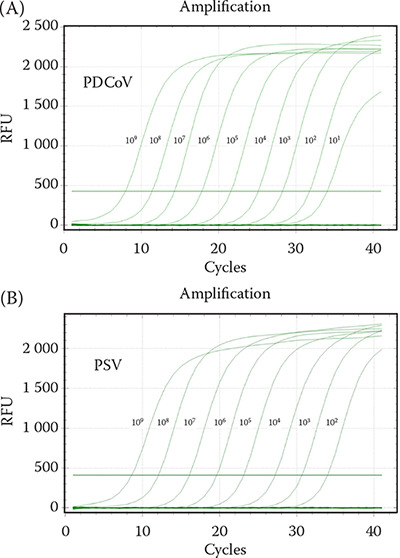
The sensitivity analysis of the duplex real-time qPCR assay for PDCoV and PSV (A) The sensitivity analysis of the duplex real-time qPCR assay for PDCoV. (B) The sensitivity analysis of the duplex real-time qPCR assay for PSV

The ten-fold serially diluted standard plasmids (10^9^ to 10^0^ copies μl^–1^) of PDCoV and PSV were amplified by the optimised duplex real-time qPCR to assess the sensitivity of the duplex real-time qPCR. According to the amplification curves (Figure 4), the minimum detection of PDCoV and PSV was 1.0 × 10^1^ copies μl^–1^ and 1.0 × 10^2^ copies μl^–1^, respectively.

### Specificity of the duplex SYBR Green I real-time qPCR

**Figure 5:**
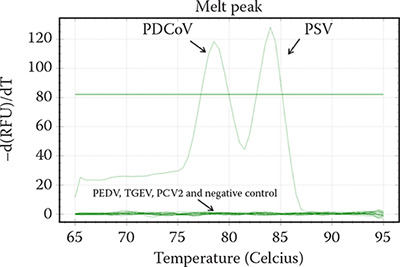
The specificity analysis of the duplex real-time qPCR assay

For the specificity, the nucleic acids of PDCoV, PSV, PEDV, TGEV and PCV2 were simultaneously detected by the duplex SYBR Green I real-time qPCR and the melting curves analysis showed that there were no specific melting peaks for PEDV, TGEV and PCV2, and the specific Tm values for PDCoV and PSV were about 78.5 °C and 84 °C, respectively (Figure 5).

### Reproducibility of the duplex SYBR Green I real-time qPCR

Furthermore, the 1.0 × 10^8^, 1.0 × 10^6^, and 1.0 × 10^4^ copies μl^–1^ for PDCoV and PSV of the standard plasmids were detected in parallel under the same condition to assess the inter- and intra-reproducibility. The intra-assay coefficient of variations (CVs were between 0.23% and 0.69%, and the inter-assay CVs ranged from 0.24% to 0.89%, respectively, which were all less than 1% showing great reproducibility of the duplex real-time qPCR assay (Table 2).

**Table 2:** Reproducibility of the duplex real-time qPCR for PDCoV and PSV

Pathogen	Plasmid concentration (copies μl^–1^)	Intra-assay			Inter-assay	
		mean (Ct) **±** SD	CV (%)		mean (Ct) **±** SD	CV (%)
PDCoV	1 × 10^8^	11.45 ± 0.03	0.23		11.50 ± 0.04	0.34
	1 × 10^6^	17.99 ± 0.12	0.69		18.04 ± 0.06	0.35
	1 × 10^4^	25.03 ± 0.12	0.47		25.08 ± 0.06	0.24
PSV	1 × 10^8^	12.03 ± 0.07	0.59		11.98 ± 0.11	0.89
	1 × 10^6^	19.55 ± 0.06	0.31		19.52 ± 0.09	0.48
	1 × 10^4^	27.08 ± 0.13	0.47		27.04 ± 0.10	0.36

Ct = cycle threshold; CV = coefficient of variation; PDCoV = porcine deltacoronavirus; PSV = porcine sapelovirus; SD = standard deviation

### Clinical application of the duplex SYBR Green I real-time qPCR assay

Two hundred and three (203) intestinal and faecal samples collected from the diarrhoeal or asymptomatic pigs were tested by the singular and duplex SYBR Green real-time qPCR for PDCoV and PSV. The results showed that the infection rate of PDCoV was 20.2% (41/203), and the infection rate of PSV was 23.2% (47/203). There were 28 samples positive for both PDCoV and PSV, and the co-infection of PDCoV and PSV was 13.8% (28/203) (Table 3). The consistency of PDCoV and PSV detected by the singular and duplex real-time qPCR is 100%, indicating that the duplex real-time qPCR method established in this study is stable and reliable.

**Table 3:** Clinical application of the duplex real-time qPCR in the 203 samples

Pathogen	Sample source		Positive rate
	asymptomatic pig	diarrheic pig	
PDCoV	0	41	20.2% (41/203)
PSV	4	43	23.2% (47/203)
PDCoV + PSV	0	28	13.8% (28/203)
Negative	56	31	42.9% (87/203)

PDCoV = porcine deltacoronavirus; PSV = porcine sapelovirus; qPCR = quantitative polymerase chain reaction

### Comparison of the duplex SYBR Green I qPCR and conventional PCR assays

Moreover, conventional PCR assays were used to detect PDCoV and PSV in the 203 samples. The positive rates of PDCoV and PSV were 14.3% (29/203) and 17.2% (35/203), respectively, and the co-infection rate was 10.8% (22/203) (Table 4). Furthermore, five samples that tested positive by the SYBR Green I real-time qPCR, but tested negative by the conventional PCR were chosen for sequencing, and the results were in accord with duplex real-time qPCR assay confirming its dependability. The results showed that the conventional PCR was less sensitive than the duplex real-time qPCR, therefore, for earlier diagnosis, the duplex SYBR Green I real-time qPCR is more suitable.

**Table 4:** Comparison of the duplex real-time qPCR and the conventional PCR

Pathogen	Duplex SYBR Green-I qPCR	Conventional PCR
PDCoV	20.2% (41/203)	14.3% (29/203)
PSV	23.2% (47/203)	17.2% (35/203)
PDCoV + PSV	13.8% (28/203)	10.8% (22/203)

PCR = polymerase chain reaction; PDCoV = porcine deltacoronavirus; PSV = porcine sapelovirus; qPCR = quantitative polymerase chain reaction

### Comparison of duplex SYBR Green I qPCR and TaqMan qPCR assays

In addition, to further verify the accuracy of the duplex SYBR Green I qPCR assay, 203 samples were tested by the TaqMan qPCR assays. The detection results are listed in Table 5, showing a 100% coincidence between the duplex SYBR Green I qPCR and the TaqMan qPCR assays, which indicated that the method developed in this study can be used as a clinical detection tool for PDCoV and PSV, providing convenience for the simultaneous detection of the two pathogens.

**Table 5:** Comparison of the duplex real-time qPCR and the TaqMan qPCR

Detecting methods	PDCoV	PSV	PDCoV + PSV	Coincidence rate (%)
Duplex SYBR Green-I qPCR (PDCoV/PSV)	20.2% (41/203)	23.2% (47/203)	13.8% (28/203)	100%
TaqMan qPCR (PDCoV)	20.2% (41/203)	–	13.8% (28/203)	
TaqMan qPCR (PSV)	–	23.2% (47/203)		

PDCoV = porcine deltacoronavirus; PSV = porcine sapelovirus; qPCR = quantitative polymerase chain reaction

## DISCUSSION

PDCoV mainly spreads vertically, causing severe diarrhoea, emesis, dehydration and acute atrophic enteritis in neonatal piglets. Since PDCoV was discovered in 2012, it has rapidly spread globally, causing great financial losses to the global pig industry and has the risk of cross-species infection (Luo et al. 2021), making prevention and control more difficult. In addition, the prevalence of PDCoV in the Henan province of China was 23.49%, and the positive rate of PDCoV in suckling piglets was 36.43% (Zhang et al. 2019), which indicates that the epidemiology surveillance of PDCoV is very important and developing a detection method for PDCoV with high sensitivity and specificity is urgent. PSV can infect pigs of any age group, especially weaned piglets, with clinical symptoms of enteritis, pneumonia, poliomyelitis, and reproductive disorders (Donin et al. 2014; Son et al. 2014; Arruda et al. 2017; Masuda et al. 2018; Li et al. 2019a). Moreover, in an epidemiological survey of PSV in Chinese pig herds, the positive rate of PSV was 44.93% in the diarrhoea samples. Even in 47 asymptomatic samples, 17 samples were detected positive for PSV and the positive rate was 27.66%, indicating that PSV is a common pathogen of diarrhoea and has a high infection rate in Chinese pig herds (Li et al. 2019b).

In order to detect the two viruses, many methods have been developed. At present, the most common detection method is conventional PCR. However, the conventional PCR assay is time-consuming with a low sensitivity rate. The detection limits of conventional PCRs for PDCoV and PSV are 1.40 × 10^2^ copies μl^–1^ and 10^4^ copies μl^–1^, respectively (Jiang et al. 2019; Ding et al. 2020). The low sensitivity can increase the false negative rate. Therefore, the conventional PCR assay is not suitable as a diagnostic tool for large sample detection. Comparing the TaqMan quantitative real-time PCR assay and SYBR Green I qPCR assay, the detection limit of the TaqMan qPCR assay for PSV is 1 × 10^2^ copies μl^–1^ (Kumari et al. 2020), which is similar to the assay developed in this study. While the detection limit of the TaqMan qPCR assay for PDCoV is 1 × 10^2^ copies μl^–1^ (Pan et al. 2020), which is less sensitive than that of our assay. Even though the TaqMan qPCR assay and SYBR Green I qPCR assay have high sensitivity, the latter was less expensive.

At present, there is no vaccine against PDCoV and PSV, thus epidemics of PDCoV and PSV should be prevented from the perspective of biosafety. A better feeding and management procedure and a better detection method can effectively reduce the occurrence of the diseases. However, as far as we know, there is no duplex real-time qPCR assay to simultaneously detect PDCoV and PSV. In recent years, the infection rate of PDCoV and PSV in pigs is on the rise, and co-infections with other porcine viruses are common (Prodelalova 2012). The co-infection rate of PDCoV and PSV was 13.04% (12/92) according to Ding et al. (2021). It is necessary to develop a method to simultaneously detect the two viruses for further molecular epidemiological investigations.

In this study, a duplex real-time qPCR assay was successfully developed to simultaneously detect PDCoV and PSV. The two viruses were easily distinguished by their Tm values of PDCoV (78.5 °C) and PSV (84.0 °C). Moreover, the detection limits of the assay were 1 × 10^1^ copies μl^–1^ for PDCoV and 1 × 10^2^ copies μl^–1^ for PSV. For clinical application value, two hundred and three (203) intestinal and faecal samples were collected from diarrhoeal or asymptomatic pigs to detect PDCoV and PSV via duplex SYBR Green real-time qPCR, conventional PCR and TaqMan qPCR methods and the results were consistent, verifying the reliability of the developed method in this study. The results indicated that the co-infection rate of PDCoV and PSV was 13.8%, suggesting that the co-infection of PDCoV and PSV should not be ignored. Compared with the conventional PCR assay, the SYBR Green I-based real-time qPCR assay is much more sensitive and costs less than the TaqMan real-time qPCR, which is more suitable as a detection tool for intensive pig farms. In this study, the positive rates detected by the duplex real-time qPCR assay for PDCoV and PSV were 20.2% and 23.2%, respectively, which was more sensitive than that of the conventional PCRs with a positive rate of PDCoV (14.3%) and PSV (17.2%).

Furthermore, four of the sixty samples collected from asymptomatic pigs tested positive for PSV and the sequencing results were correct. Two of the four positive samples were from a 17-day gestation sow and a 99-day gestation sow. The results suggest that sows may develop a strong immunity to PSV and it is difficult for feeders to notice it from the symptoms. However, piglets have weak immune systems and can easily be infected with PSV through sows. Once transmitted from sows to piglets, it will cause diarrhoea and other symptoms, which will increase the mortality of piglets and bring huge economic losses to pig farms. Therefore, the regular PSV detection of sows and piglets is essential. Therefore, it is necessary to establish a rapid, accurate, and low-cost method for the periodic detection of PDCoV and PSV in large groups.

In conclusion, we have developed a duplex SYBR Green I real-time qPCR method for the detection of PDCoV and PSV. The results show that the method had high sensitivity, strong specificity, and good repeatability, which can be used for clinical detection. Moreover, this approach provides a rapid and reliable laboratory diagnostic tool for the investigation and clinical diagnosis of PDCoV and PSV co-infections.
